# A Machine Learning Framework for Detecting COVID-19 Infection Using Surface-Enhanced Raman Scattering

**DOI:** 10.3390/bios12080589

**Published:** 2022-08-02

**Authors:** Eloghosa Ikponmwoba, Okezzi Ukorigho, Parikshit Moitra, Dipanjan Pan, Manas Ranjan Gartia, Opeoluwa Owoyele

**Affiliations:** 1 Department of Mechanical and Industrial Engineering, Louisiana State University, Baton Rouge, LA 70803, USA; eikpon1@lsu.edu (E.I.); oukori1@lsu.edu (O.U.); 2 Department of Pediatrics, Center for Blood Oxygen Transport and Hemostasis, University of Maryland Baltimore School of Medicine, Baltimore, MD 21201, USA; pxm5519@psu.edu (P.M.); dipanjan@psu.edu (D.P.); 3 Department of Nuclear Engineering, The Pennsylvania State University, University Park, PA 16802, USA

**Keywords:** surface-enhanced Raman spectroscopy, machine learning, COVID-19, Gaussian processes

## Abstract

In this study, we explored machine learning approaches for predictive diagnosis using surface-enhanced Raman scattering (SERS), applied to the detection of COVID-19 infection in biological samples. To do this, we utilized SERS data collected from 20 patients at the University of Maryland Baltimore School of Medicine. As a preprocessing step, the positive-negative labels are obtained using Polymerase Chain Reaction (PCR) testing. First, we compared the performance of linear and nonlinear dimensionality techniques for projecting the high-dimensional Raman spectra to a low-dimensional space where a smaller number of variables defines each sample. The appropriate number of reduced features used was obtained by comparing the mean accuracy from a 10-fold cross-validation. Finally, we employed Gaussian process (GP) classification, a probabilistic machine learning approach, to correctly predict the occurrence of a negative or positive sample as a function of the low-dimensional space variables. As opposed to providing rigid class labels, the GP classifier provides a probability (ranging from zero to one) that a given sample is positive or negative. In practice, the proposed framework can be used to provide high-throughput rapid testing, and a follow-up PCR can be used for confirmation in cases where the model’s uncertainty is unacceptably high.

## 1. Introduction

Coronavirus disease (COVID-19), caused by the SARS-CoV-2 virus, is a viral infection that is primarily spread when people breathe in air contaminated with the virus, either through aerosolized particles or droplets expelled by infected persons [1]. Due to the severity of this disease and its rapid spread, there is a pressing need for high-throughput and quick and reliable testing methods. To enable the better management of patients and implementation of proactive steps to limit transmission rates, timely identification of SARS-CoV-2 infection in affected patients is essential [2]. Several COVID-19 testing methods have been developed to diagnose the disease, including the polymerase chain reaction test (PCR) [2], antigen-based tests [3], and serological enzyme-linked immunosorbent assay (ELISA) [4]. However, while the PCR is the most accurate and frequently used as a benchmark against which other tests are measured, it has certain drawbacks, including being time-consuming [5] and being a sample-dependent method with a high false-negative ratio [6]. This makes it unsuitable for situations where rapid medical and personal decisions need to be made. Furthermore, the ELISA approach is based on immunoassay sensitivity and requires accurate coupling between an enzyme-coupled antibody and numerous viral-specific antigens [7]. In response to the limitations, a number of previous studies have utilized Raman spectroscopy (RS) for diagnoses. In their study, Desai et al. used Raman spectroscopy for SARS-CoV-2 detection through the saliva [8]. In their research, Yin et al. proposed a method of detecting COVID-19 based on Raman spectroscopy, concluding that it is a safe and effective method for the detection of the disease [6]. In another study, Carlomagno et al. developed a Raman-based classification model that was able to distinguish COVID-19 patients with an accuracy range of about 89–92% [9].

Raman spectroscopy is a non-invasive diagnostic method that can reveal chemical and biochemical information embedded in cells [10]. It is based on the interaction of photons in an incident beam with a material’s chemical bonds. Raman (or inelastic) scattering occurs when light photons make direct contact with the sample. This causes molecular excitations that lead to vibrations. The difference in energy between the incident and scattered photons correlates to the energy of molecular vibrations in the sample, and these show up as spikes in the resulting spectra [11]. The spectra provide information about the presence of some biochemicals in the sample based on the intensity of the spikes. One limitation, however, of Raman spectroscopy is its low sensitivity arising from low signals. In response to this issue, the Raman signal is sometimes amplified using plasmonic particles in a process known as surface-enhanced Raman scattering (SERS). The spectral information from Raman spectroscopy (and SERS) has been used in previous studies for tasks such as detecting the occurrence of cancerous cells [12] hepatitis B virus [13], tuberculosis [14], dengue virus [15], and the influenza virus A [16], to mention a few.

The spectra of heterogeneous bio-systems, including many biomolecules, such as cells, tissues, and biofluids, are complicated and high dimensional [8]. A Raman spectrum is typically made up of 500–3000 features (i.e., Raman intensities) and most datasets contain very few samples [17]. As a result, to extract useful information and gain a deeper understanding, it is often necessary to pre-process the data and reduce its dimensionality. Many data preprocessing and dimensionality reduction techniques have been applied to Raman spectroscopy data, such as non-negative matrix factorization (NNMF), principal component analysis (PCA), variational autoencoders (VAEs), and uniform manifold approximation and projection (UMAP) [11,12,17,18,19,20]. What these techniques have in common is that they are used to map data from high-dimensional input spaces to lower-dimensional subspaces. Within the low-dimensional subspace, the number of variables that defines the dataset is reduced, yet the majority of the variance in the original dataset remains retained. The dimensionality reduction helps alleviate the problem of the curse of dimensionality, which as just described, is a frequent difficulty that arises in Raman spectral data analysis. In their research, Desai et al. [8] used PCA to reduce the features from Raman spectral data to two principal components, covering 76% of the total variance of the dataset.

The reduced set of variables obtained from the dimensionality reduction techniques as described above is often fed as inputs to various machine learning classifiers. Vidales et al. [21] showed in their work that PCA and support vector machine (SVM) algorithms correctly distinguished between wild and mutant types of the p53 cancer biomarker with an accuracy of 94%. Bovenkamp et al. [22] achieved 93% accuracy in distinguishing between low- and high-grade lesions using PCA followed by KNN, demonstrating that RS can be efficiently integrated with machine learning as a preferred strategy for diagnosing cancer. Another study examined RS’s capability to distinguish between benign lesions and malignant cancer samples, which were gathered from 20 different donors. On the spectrum dataset, a number of chemometric techniques were used. Principal component analysis-linear discriminant analysis (PCA-LDA), principal component analysis-quadratic discriminant analysis (PCA-QDA), and partial least squares-discriminant analysis (PLS-DA) all produced classification results with greater than 80% sensitivity and specificity, while principal component analysis-support vector machines (PCA-SVM) produced classification results with greater than 90% sensitivity and specificity [23]. In order to reduce the effects of high feature dimension and noise in RS data, He et al. utilized variational autoencoders. The data was then classified using a variety of machine learning techniques, with Gaussian Naïve Bayes achieving the best accuracy [19].

The aim of this paper was to introduce a methodology for high-throughput and rapid COVID-19 detection. Besides an accurate model, it is also of primary concern to have a reliable estimate of the model’s uncertainties. In other words, we sought to obtain a model that could answer the following question: how much confidence can we place in a predicted negative or positive diagnosis? To this end, we employed Gaussian processes for COVID-19 diagnoses, using data obtained from SERS. Using a Bayesian framework as its foundation, Gaussian processes belong to a class of non-parametric techniques. The underlying probability densities are presupposed to have a prior distribution, which ensures certain smoothness properties. Given a sample, the GPC approach used in this paper provided a robust positive–negative probability for each class, from which the uncertainty associated with the sample could be estimated. We also evaluated the dimensionality reduction effects of PCA, a linear dimensionality reduction technique, and UMAP, a nonlinear dimensionality reduction technique. Grid search and cross-validation were used to get the optimal hyperparameter for building the classifier due to the small size of our dataset. Finally, we discuss the significance of our results and close the paper with some concluding remarks.

## 2. Materials and Methods

### 2.1. Data Collection and Preprocessing

Standard commercial kits were used to extract and purify RNA from the clinical samples. Antisense oligonucleotides (ASOs) were designed based on the whole genome sequence of SARS-CoV-2 [24,25,26,27,28,29,30,31,32]. The ASOs were 20 nucleotides in length. Four ASOs were used to target the genes (N and E gene) [29]. The thiolated ASOs were used to cap citrate-stabilized gold nanoparticles (AuNPs). For the Raman experiments, 2 µL of Au-ASO NPs (concentration of 2 × 10^11^ particles/mL) were mixed with 2 µL of SARS-CoV-2 RNA (concentration ranging from 1 fg/mL, i.e., 63 copies/mL to 1 μg/mL, i.e., 63 × 10^9^ copies/mL) by gentle pipetting a few times on an ice rack [12,30,33,34]. An amount of 2 µL of this mixture was drop-casted on a clean stainless-steel slide and then allowed to air-dry at room temperature. Immediately after, Raman spectra of the dried spots were recorded using a Renishaw inVia Reflex Raman Spectroscope. In the scenario, where Raman spectra were acquired directly from the clinical samples and without the extraction of RNA, the samples were first added with lysis buffer containing guanidine isothiocyanate at a 2:1 *v/v* ratio. 2 μL of these lysed samples were then added with 2 μL of Au-ASO NPs (concentration of 2 × 10^11^ particles/mL) and mixed adequately as discussed earlier. The Raman spectra of AuNP, SARS-CoV-2 RNA, and SERS spectra of Au-ASO, Au-ASO-SARS-CoV-2 RNA are provided in the Supplementary Information (Figures S1–S3).

**Figure 1:**
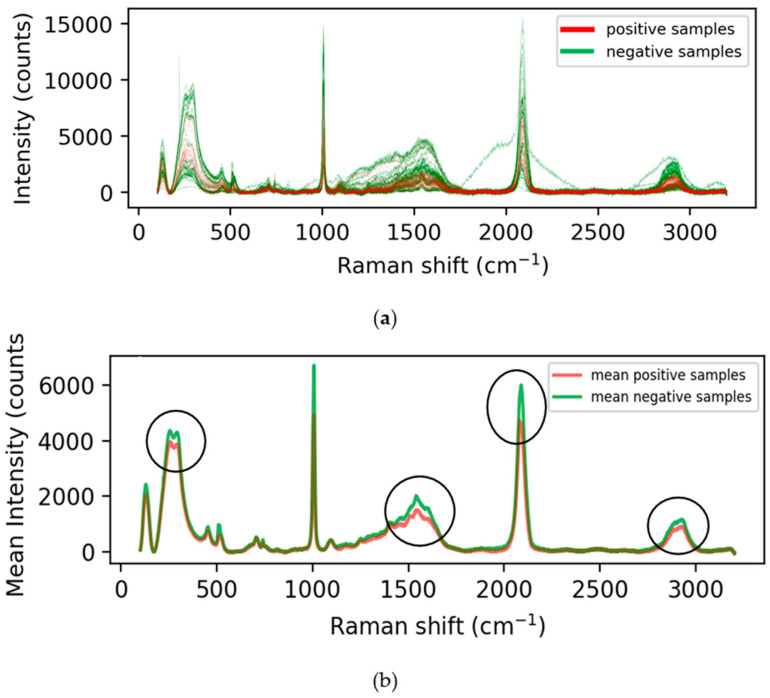
(**a**) Positive and negative SERS Spectral (**b**) Mean positive and negative SERS.

Raman experiments were performed using a laser with excitation wavelengths of λ = 785 nm, grating = 1200, power = 10%, exposure time = 10 s, objective = 50X long working distance (LWD). At least ten spectra were acquired from each sample for statistical analysis in the range of 100–3200 cm^−1^. Raman images were acquired in StreamHR image acquisition mode using a step size (resolution) of 200 × 200 µm for clinical samples and 70 × 70 µm for RNA samples. During the Raman imaging, the exposure time was set to be 0.5 s. Renishaw WiRE 4.4 was used for data processing and analysis of Raman signals and images. For baseline correction, we used intelligent fitting of WiRE 4.4 with a polynomial order of 11 and noise tolerance of 1.5. Subsequent data processing was also performed by OriginLab 2018 [28]. We utilized SERS data collected from 20 patients at the University of Maryland, with the positive–negative labels obtained using Polymerase Chain Reaction (PCR) testing. The data collected were evenly balanced, containing a 50–50 split of positive and negative samples. The raw SERS spectra have intensities collected over 3062 Raman shifts, amounting to 3062 features. The entire positive and negative spectra as well as the mean spectra have been plotted in Figure 1a,b.

The positive and negative SERS spectra were stacked together in an array, with the samples in the rows and intensity for a given Raman shift on the columns. This resulted in a dataset $X\in R^{m\times n}$, where *m* is the number of samples, and *n* is the number of Raman shifts. Before performing dimensionality reduction, the data was normalized between 0 and 1 to avoid low-magnitude features being weighted unfairly.

### 2.2. Dimensionality Reduction

#### 2.2.1. Principal Component Analysis (PCA)

Principal component analysis (PCA) is a dimensionality reduction strategy that takes advantage of correlations between existing variables to produce a new collection of uncorrelated features known as principal components (PCs). It is an unsupervised technique that projects a high-dimensional data matrix onto a lower-dimensional subspace by performing a linear transformation. The high dimensional data is reduced to smaller primary components that are orthogonal and uncorrelated, with each successive component chosen based on the direction of maximum variance. In other words, it decreases dimensionality while maximizing the retained variance [17]. Dimensionality reduction is important for this dataset because of the large number of Raman shifts and the relatively small data samples. To build an ML model that performs well on this dataset and minimizes overfitting, dimensionality reduction is key.

#### 2.2.2. Uniform Manifold Approximation and Projection (UMAP)

PCA is a linear approach and is, therefore, inefficient when dealing with data that contains nonlinear structures. Therefore, we also performed dimensionality reduction using UMAP [20], a nonlinear manifold learning technique, and compared the results with those obtained from PCA. UMAP is a relatively recent dimensionality reduction method that assumes the available data samples are evenly distributed across a topological space or manifold, which can be approximated from these finite data samples and mapped to a lower-dimensional space [20]. This technique excels at capturing nonlinear structures in high-dimensional data, particularly at a local level, which means that if two points are close together in the high-dimensional space, they are likely to be close together in the low-dimensional embedding as well. The UMAP has different hyperparameters that can impact the lower dimensional embeddings created, described below.

- The number of neighbors: This controls the focus on local or global structure in the data. Lower values of this parameter force the UMAP to focus on a very local structure, while the higher values will make the UMAP focus on larger or global structures.
- The minimum distance: This parameter governs how closely UMAP can pack points together. Lower numbers indicate that the points will be tightly clustered and vice versa.
- The number of components: This determines the dimensionality of the low-dimensional space.

### 2.3. Machine Learning Classification

In this study, a supervised learning approach—Gaussian Process Classifier (GPC) was employed to learn the appropriate labels for the samples. Gaussian processes are non-parametric methods based on the Bayesian methodology. It yields probabilistic classification, with each prediction representing a probability over each class. The choice of GPC stems from the fact that their prediction is based on more a rigorous treatment of probability when compared to other approaches. Because of their probabilistic output, uncertainty estimates can be calculated, this would enable one to assess the degree to which the prediction can be relied upon. A high confidence value would indicate that the predicted class is likely accurate, but a low confidence score would be inconclusive, and would perhaps, indicate that confirmation via a PCR test is advisable. In general, the quantification of uncertainties is important in medical diagnostic applications because of the potential significance of a misdiagnosis. Therefore, a model that predicts a confidence score alongside its predictions is vital to reducing the rate of the wrong diagnosis. The PCA and GPC models were created using Scikit-learn v1.0.2 [35], while the UMAP reduction was obtained using the open-source UMAP library on GitHub [20].

A *k*-fold cross-validation approach, where the dataset was partitioned into 10 parts (or folds), was employed. An iterative procedure was used to loop over the folds, where the *k*th fold was used for validation at a given iteration, while other folds were used for model training. In this way, the model’s performance for various subsets of the dataset can be determined. The performance metrics used in this study were accuracy, precision, recall, F1-score, and area under the curve (AUC). Accuracy measures the ratio of samples that were correctly labeled to the total number of samples in the datasets. Accuracy is often reliable when the dataset has balanced classes, which was the case in this study. Precision is the ratio of accurately predicted positive observations to the total number of predicted positives, while recall (also known as sensitivity) is the ratio of the number of correctly predicted positive observations to the number of actual positive occurrences. The F1-score is the harmonic mean of both precision and recall. A model with a high F1 score indicates high precision and recall values.

## 3. Results and Discussion

**Figure 2:**
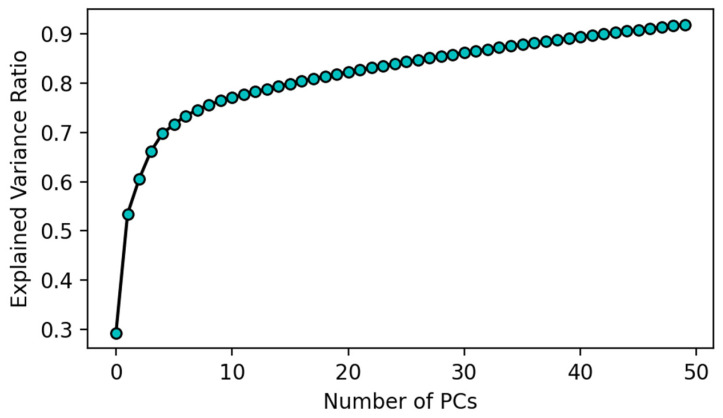
Explained Variance Ratio Plot for PCA.

Figure 1 and Figure 2 show the SERS spectral and mean intensities of all the positive and negative samples, respectively. The mean intensity plot shows a significant distinction between the positive and negative samples, with a Raman shift of 1300–1600, 2100–2200 and 2800–3000 cm^−1^. As mentioned earlier, each spectrum has 3062 entries, which can be interpreted as features with varying intensity levels. Many of these features are redundant and do not contribute to the separation of the classes. Therefore, we performed dimensionality reduction using linear (PCA) and nonlinear (UMAP) dimensionality reduction techniques, as described in Section 2.

**Figure 3:**
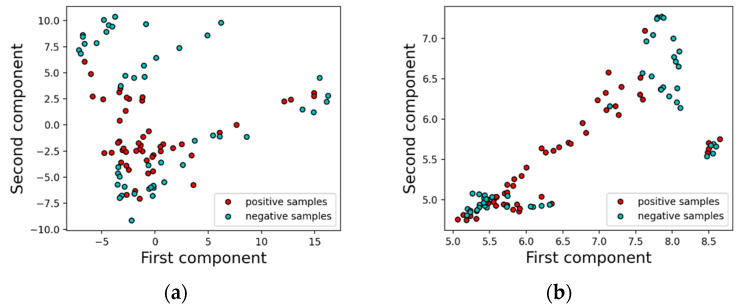
(**a**) 2D scatter plot of first and second principal components from PCA (**b**) 2D scatter plot of first and second components from UMAP.

Figure 2 shows the ratio of the variance captured as a function of the number of retained variables. A higher ratio of variance captured means that most of the information in the dataset has been captured by the retained PCs, and therefore, it is desirable to capture a high variance ratio in the first few leading PCs. From the figure, we can see that over 90% of the variance in the data is captured by using just 50 features. On the other hand, using only two principal components only captures 55% of the variance, or put differently, loses 45% of the variance in the original dataset. For visualization purposes, the first two components for both PCA and UMAP were plotted for comparison in Figure 3a,b, respectively, with red symbols depicting positives and cyan symbols depicting negative cases. In both cases, some degree of separation can be seen, but significant regions of overlap between positive and negative cases exist. For instance, for PCA in Figure 3a, positive cases are concentrated between PC2 = −2.5 and 2.5, but below 2.5, positive and negative cases co-exist in close proximity. The imperfect separation of the two classes should come as no surprise since only 55% of the variance in the data was captured by two PCs. Some degree of overlap can also be seen in Figure 3b, suggesting that even when nonlinear projections are employed, the dimensionality required to find reasonable separation boundaries between positive and negative cases is higher than two.

**Figure 4:**
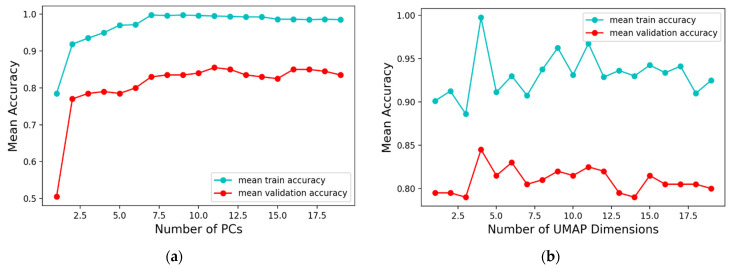
(**a**) Comparison of different number of principal components from PCA with the model’s mean accuracy across 10 folds (**b**) Comparison of different number of components from UMAP with the model’s mean accuracy across 10 folds.

Therefore, to select the optimum number of reduced variables that enable easy separation of the classes, various numbers of components (ranging from 2 to 20) were retained and used as input features for the classifier. Figure 4a shows that for PCA, the mean *k*-fold accuracy (computed by averaging the performance across all folds) increased as the number of PCs increased, and there was no significant increases in the mean validation accuracy after 11 PCs. Thus, 11 was selected as the number of PCs that gives the model satisfactory information to classify our dataset. Figure 4b shows the mean accuracy versus UMAP number of dimensions; the average *k*-fold accuracy peaks at four dimensions and gradually declines when increased from this point. Therefore, four was selected as the optimal number of reduced dimensions for the model. Furthermore, a grid search process was used to find the optimal number of neighbors and minimum distance. Using the accuracy score as the search objective, optimal values of 10 and 0.1 were found for the number of neighbors and minimum distance, respectively.

The GPC model was tested separately for the reduced set of variables obtained from PCA and UMAP. The kernel (or covariance function) selected for both the PCA-GPC and UMAP-GPC models was the Matérn kernel, which requires the selection of a length scale, *l*, and a smoothness-controlling parameter, ν. For the PCA case, *l* was chosen as 6.51, while ν was 1.5. For the UMAP-GPC the parameters chosen were *l* = 0.646 and ν = 1.5. The model’s accuracy, precision, recall, F1-score, and AUC have been summarized in Table 1 and Table 2. The table shows that UMAP-GPC and PCA-GPC result in similar accuracies and F1 scores, while for precision and recall, the UMAP-GPC model produces higher values when compared to PCA-GPC.

**Figure 5:**
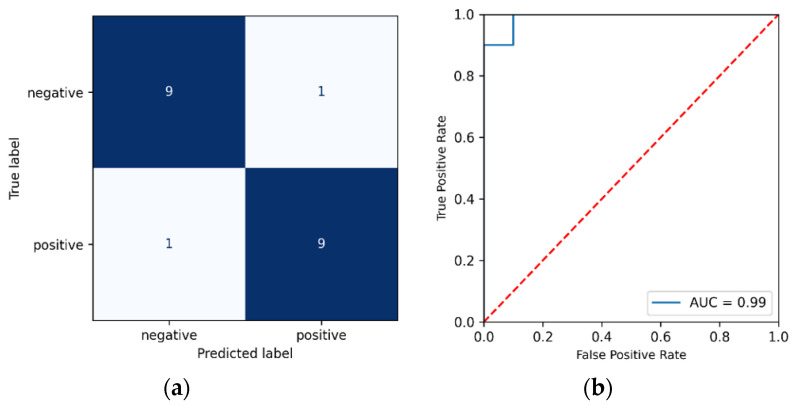
(**a**) Confusion Matrix for Gaussian Process Classifier with 7 principal components (**b**) Area Under Curve Receiver Operating Characteristics (AUC_ROC) for Gaussian Process Classifier with 7 principal components.

**Figure 6:**
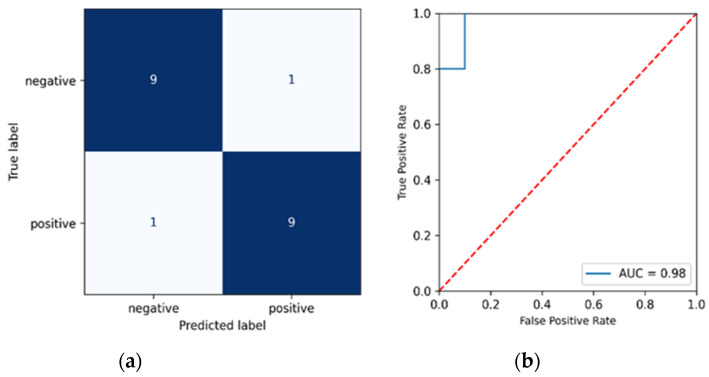
(**a**) Confusion Matrix for Gaussian Process Classifier with 4 UMAP components (**b**) Area Under the Receiver Operating Characteristics (AUC_ROC) curve for Gaussian Process Classifier with 4 UMAP components.

In order to further evaluate the model’s performance, the AUC (Area Under the Curve) ROC (Receiver Operating Characteristics) curve was also created. AUC represents the degree of distinction between classes in our data, i.e., how well our model can predict each individual class correctly. The greater the AUC, the better the model distinguishes between positive and negative classes. Furthermore, Table 1 and Table 2 show the mean AUC to be 0.941 and 0.929 for the PCA and UMAP models, respectively, indicating that the model is able to predict the class labels with minimum error. Figure 5a,b shows the ROC of one-fold chosen randomly from the 10-folds (fold 9 for PCA-derived variables), alongside the confusion matrix. We see that the model predicted 9 out of the 10 negative classes correctly and 9 out of the 10 positive classes when using the PCA-derived variables. Similarly, Figure 6a,b shows the ROC and confusion matrix for fold 10 based on the UMAP-derived variables. As in Figure 5a,b for the PCA-derived variables, we see that the model correctly predicts 90% of all positive and negative samples.

Table 3 and Table 4 show the prediction probability score and uncertainty for the test predictions. Given a random variable *X* with observations {*x*_1_, *x*_2_, …, *x_n_*}, the uncertainty is estimated using the Shannon entropy, defined as

(1) $$H\left(X\right)=-\overset{n}{\underset{i=1}{\sum }}P\left(x_{i}\right)logP\left(x_{i}\right)$$

**Figure 7:**
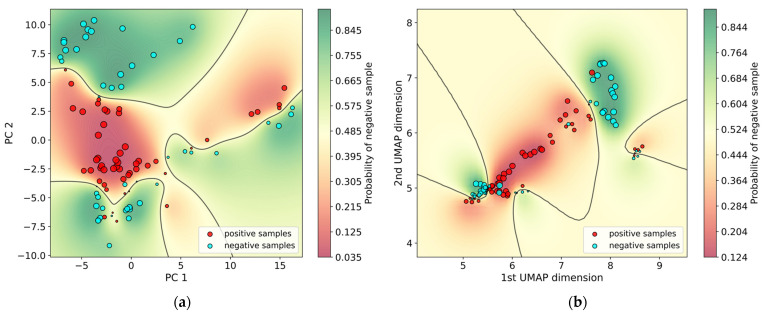
(**a**) GPC model decision boundary plot with uncertainty estimation for PCA (**b**) GPC model decision boundary plot with uncertainty estimation for UMAP.

The Shannon entropy measures the amount of information in *X* and ranges from zero to one [36], with a value of one indicating high uncertainties and a value of zero indicating high confidence. It is desirable that a model produces high Shannon entropies (low confidence) when it misclassifies a sample, and low uncertainties when it produces an accurate label. Table 3 and Table 4 show that this is the case with both PCA-GPC and UMAP-GPC—the uncertainty estimates for inaccurate predictions are relatively high, compared to the rest. In Figure 7, we also visualized the model’s decision boundary for a special case with only two components, due to difficulties of visualization with higher dimensions. In the figure, the size of the circle point correlates with the prediction confidence (i.e., small symbols indicate low confidence). The plots show that points closer to the decision boundary and misclassified points have very small sizes, implying low confidence and high uncertainty. As mentioned above, this is useful because when the classifier misclassifies a prediction, it signals that the prediction was highly uncertain. In practice, a cutoff uncertainty level can be recommended, above which the result from the GPC model cannot be trusted and the patient is referred for further testing.

## 4. Conclusions

In this paper, we proposed an approach based on dimensionality reduction and a probabilistic Gaussian process classification model to classify COVID-19 data obtained from SERS. Both linear PCA-based dimensionality reduction and a stochastic nonlinear UMAP dimensionality reduction technique were compared. A hyperparameter search process revealed the optimal number of dimensions for UMAP was four, while PCA performed best when 11 principal components were utilized. Using the optimal number of dimensions, both techniques resulted in similar model accuracies and comparable ROC-AUC curves. Even though the dataset used in this study was relatively sparse, the results obtained are promising. For PCA, the leave-out validation accuracy was 85.5%, while the average precision was 91.5%. Similarly, UMAP-based reduction resulted in a mean accuracy and precision of 85.5 and 92.5%, respectively. It is reasonable to assume that the model’s performance would be substantially better if a larger dataset was used for training. Since GPC provides a probability (ranging from 0–100%) that a given sample is positive or negative, rather than offering hard class labels, we were able to estimate the uncertainty of the model’s predictions by computing the Shannon entropies. For both PCA- and UMAP-based models, we showed that the uncertainties for misclassified samples were the highest (1.0). In practice, when such samples with high uncertainties are detected, patients can be recommended for further PCR testing to confirm or disprove the diagnosis. Furthermore, the proposed framework can be utilized to provide high throughput testing in settings where speed and reliability are critical. The proposed approach is also promising for use in miniaturized point of care devices, as opposed to the use of specialized laboratory testing as done in PCR. A spectra containing specific biochemical markers can be collected in as little as a few minutes, while the machine learning inference (PCA or UMAP, followed by GPC) to predict the occurrence of a positive or negative sample can be performed in as little as a few seconds. Overall, the result from this study demonstrates the potential of SERS and machine learning algorithms to effectively diagnose COVID-19 infection with an uncertainty measure to inform medical decisions.

## Figures and Tables

**Table 1:** Results from the 10-fold validation of GPC model with 7 PCs.

Folds	Accuracy	Precision	Recall	F1 Score	ROC_AUC
Fold 1	0.800	1.000	0.600	0.750	0.920
Fold 2	0.900	0.900	0.900	0.900	0.980
Fold 3	1.000	1.000	1.000	1.000	1.000
Fold 4	0.750	0.727	0.800	0.762	0.860
Fold 5	0.700	0.750	0.600	0.667	0.810
Fold 6	0.950	1.000	0.900	0.947	0.970
Fold 7	0.850	1.000	0.700	0.824	0.920
Fold 8	0.800	0.875	0.700	0.778	0.950
Fold 9	0.900	0.900	0.900	0.900	0.990
Fold 10	0.900	1.000	0.800	0.889	0.980
Mean	0.855	0.915	0.740	0.842	0.941

**Table 2:** Result from 10-Fold validation of GPR model with 4 UMAP Dimensions.

Folds	Accuracy	Precision	Recall	F1 Score	ROC_AUC
Fold 1	0.800	1.000	0.600	0.750	0.920
Fold 2	0.900	0.900	0.900	0.900	0.980
Fold 3	1.000	1.000	1.000	1.000	1.000
Fold 4	0.750	0.727	0.800	0.762	0.860
Fold 5	0.700	0.750	0.600	0.667	0.810
Fold 6	0.950	1.000	0.900	0.947	0.970
Fold 7	0.850	1.000	0.700	0.824	0.920
Fold 8	0.800	0.875	0.700	0.778	0.950
Fold 9	0.900	1.000	0.800	0.889	0.900
Fold 10	0.900	1.000	0.800	0.889	0.980
Mean	0.855	0.925	0.780	0.841	0.929

**Table 3:** Test predictions, prediction probability and uncertainty for GPR model with PCA. The misclassified samples are highlighted in red.

Samples	Class 1 prob	Class 2 prob	True Class	Predicted Class	Uncertainty
1	0.500	0.500	1	0	1.00
2	0.376	0.624	1	1	0.95
3	0.245	0.755	1	1	0.80
4	0.608	0.392	0	0	0.97
5	0.196	0.804	1	1	0.71
6	0.689	0.311	0	0	0.89
7	0.822	0.178	0	0	0.68
8	0.179	0.821	1	1	0.68
9	0.732	0.268	0	0	0.84
10	0.231	0.769	1	1	0.78
11	0.257	0.743	1	1	0.82
12	0.238	0.762	1	1	0.79
13	0.341	0.659	1	1	0.93
14	0.466	0.534	0	1	1.00
15	0.760	0.240	0	0	0.80
16	0.654	0.346	0	0	0.93
17	0.870	0.130	0	0	0.56
18	0.686	0.314	0	0	0.90
19	0.463	0.537	1	1	1.00
20	0.764	0.236	0	0	0.79

**Table 4:** Test predictions, prediction probability and uncertainty for GPR model with UMAP. The misclassified samples are highlighted in red.

Samples	Class 1 prob	Class 2 prob	True Class	Predicted Class	Uncertainty
1	0.707	0.293	0	0	0.87
2	0.760	0.240	0	0	0.80
3	0.361	0.639	1	1	0.94
4	0.840	0.160	0	0	0.63
5	0.480	0.520	1	1	1.00
6	0.696	0.304	0	0	0.89
7	0.777	0.223	0	0	0.77
8	0.832	0.168	0	0	0.65
9	0.351	0.649	1	1	0.93
10	0.165	0.835	1	1	0.65
11	0.452	0.548	1	1	0.99
12	0.587	0.413	0	0	0.98
13	0.466	0.534	0	1	1.00
14	0.187	0.813	1	1	0.70
15	0.329	0.671	1	1	0.91
16	0.789	0.211	0	0	0.74
17	0.544	0.456	1	0	0.99
18	0.611	0.389	0	0	0.96
19	0.285	0.715	1	1	0.86
20	0.336	0.664	1	1	0.92

## Data Availability

Not applicable.

## Supplementary Materials

The following supporting information can be downloaded at: https://www.mdpi.com/article/10.3390/bios12080589/s1, Figure S1: Raman spectra of SARS-CoV-2 RNA; Figure S2: Raman spectra of Au NP and ASO; Figure S3: SERS spectra of Au-ASO, and Au-ASO-SARS-CoV-2 RNA. To compare the relative enhancement in SERS, the Raman spectra of SARS-CoV-2 is also included in the figure.
