# Supplementary material for: A Machine Learning Framework for Detecting COVID-19 Infection Using Surface-Enhanced Raman Scattering
Source: Biosensors (Basel). 2022 Aug 2;12(8):589. doi: 10.3390/bios12080589 (PMC9405612; doi:10.3390/bios12080589)
Supplement: Supplementary file 1 [file biosensors-12-00589-s001.zip › biosensors-1817255-supplementary.pdf]

Supplementary Materials

A machine learning framework for detecting COVID-19 infection using surface-enhanced Raman scattering

Eloghosa Ikponmwoba[1], Okezzi Ukorigho[1], Parikshit Moitra[2,3], Dipanjan Pan[2,3], Manas Ranjan Gartia[1],* , Opeoluwa Owoyele[1],*

[1] Department of Mechanical and Industrial Engineering, Louisiana State University, Baton Rouge, Louisiana, 70803, United States; eikpon1@lsu.edu (E.I.); oukori1@lsu.edu (O.U.); mgartia@lsu.edu (M.G.); oowoyele@lsu.edu (O.O.).

[2] Department of Pediatrics, Center for Blood Oxygen Transport and Hemostasis, University of Maryland Baltimore School of Medicine, Baltimore, Maryland 21201, USA

[3] Department of Nuclear Engineering, The Pennsylvania State University, University Park, Pennsylvania 16802, USA; pxm5519@psu.edu (P.M.); dipanjan@psu.edu (D.P.).

* Correspondence: MR: mgartia@lsu.edu; OO : oowoyele@lsu.edu

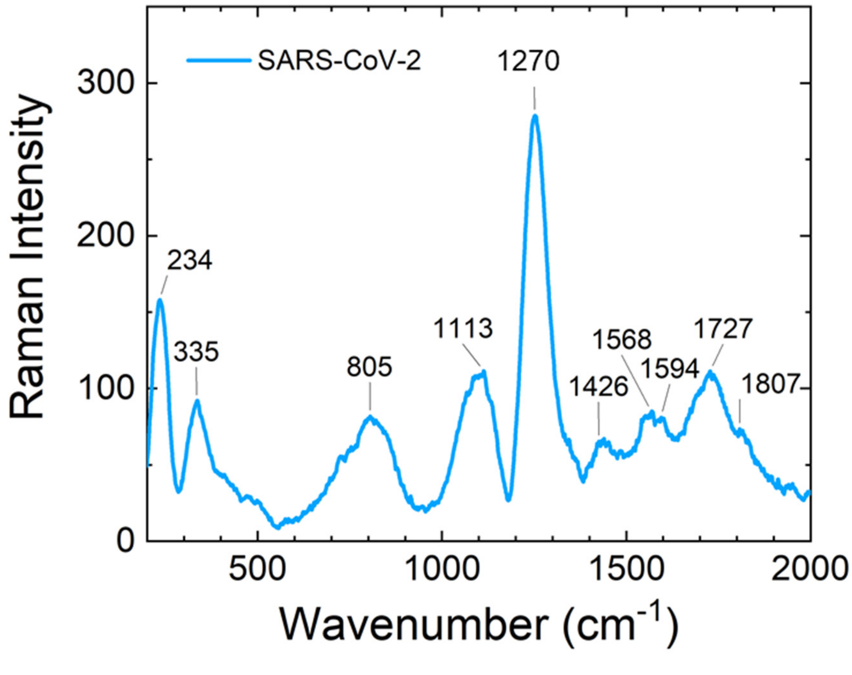

Figure S1. Raman spectra of SARS-CoV-2 RNA.

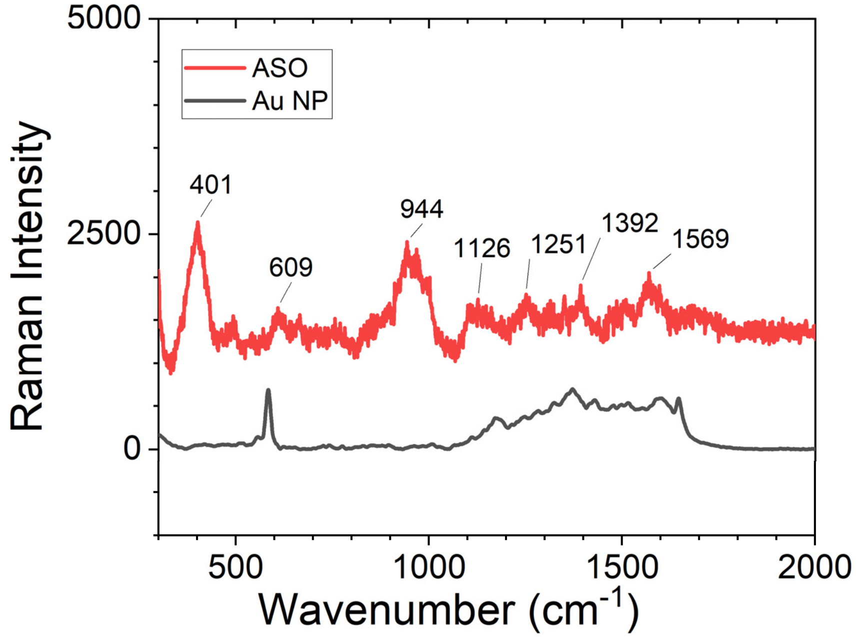

Figure S2. Raman spectra of Au NP and ASO.

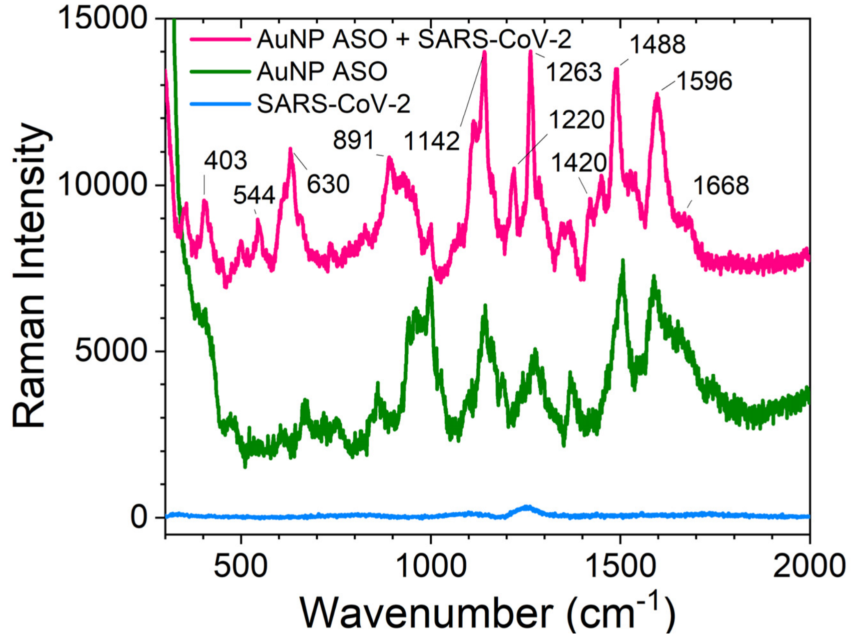

Figure S3. SERS spectra of Au-ASO, and Au-ASO-SARS-CoV-2 RNA. To compare the relative enhancement in SERS, the Raman spectra of SARS-CoV-2 is also included in the figure.
